# Atrial fibrillation: from pathogenesis to novel treatment options

**DOI:** 10.1186/s43556-025-00393-1

**Published:** 2025-12-19

**Authors:** Yi Liu, Yuwei Chen, Qiang Ren, Haoyu Zhang, Haiyuan Huang, Zhi Luo, Xingyao Xiao, Xin Chen, Juntao Li, Qian Zhang, Xiangbin Xiao

**Affiliations:** 1 Department of Cardiology Medicine, The Affiliated Hospital of Southwest Medical University, Luzhou, Sichuan 646000 China; 2 Department of Cardiology Medicine, The People’s Hospital of Jianyang City, No. 180 Hospital Road, Jianyang City, Chengdu, Sichuan Province 641400 China; 3 Chengdu Medical College, Chengdu, Sichuan 610500 China

**Keywords:** Atrial fibrillation (AF), Pathogenesis, Cryoballoon ablation, Pulmonary vein isolation (PVI), Pulsed-field ablation (PFA), Radiofrequency ablation

## Abstract

The management of atrial fibrillation (AF) is currently undergoing a significant paradigm shift, driven by a deepening understanding of pathophysiology and the urgent need to overcome the inherent safety and durability limitations of conventional thermal catheter ablation. This review provides a comprehensive update on the evolving AF landscape, systematically connecting complex pathogenetic mechanisms, from focal triggers to progressive fibrotic substrate remodeling, with emerging diagnostic and therapeutic innovations. We critically evaluate the expanding spectrum of novel treatment modalities, with an emphasis on pulsed-field ablation (PFA), detailing its biophysical basis of irreversible electroporation, superior myocardium-selective safety profile, and accumulating clinical evidence. Furthermore, the review integrates complementary advancements, including high-resolution electroanatomic mapping systems that refine substrate characterization, hybrid surgical-catheter strategies for refractory cases, and upstream pharmacologic interventions targeting disease progression. By synthesizing contemporary data on procedural workflows and efficacy outcomes from recent randomized trials, we address persisting challenges such as lesion durability and the management of non-pulmonary vein drivers. Finally, we identify critical knowledge gaps regarding long-term comparative effectiveness and propose a phenotype-guided management framework. This approach aims to leverage these diverse emerging technologies to optimize patient selection, thereby advancing the field toward safer, more durable, and truly personalized rhythm control for the growing population of patients with atrial fibrillation.

## Introduction

Atrial fibrillation (AF) stands as the most prevalent sustained cardiac arrhythmia, imposing a substantial and escalating global public health burden [1–4]. Recent analyses from the Global Burden of Disease Study 2019 highlight AF’s increasing incidence and prevalence worldwide, predicting a continued rise in affected individuals due to population aging and the growing prevalence of cardiovascular risk factors [1, 5, 6]. Beyond its characteristic symptoms ranging from palpitations and dyspnea to severe fatigue and reduced quality of life [7], AF significantly contributes to morbidity and mortality. It is an independent risk factor for ischemic stroke, increasing the risk five-fold, and is increasingly recognized for its association with heart failure, cognitive decline, and all-cause mortality [8–11]. The intricate interplay between AF and heart failure, in particular, often creates a vicious cycle that exacerbates both conditions [11].

The pathogenesis of AF is complex and continuously being elucidated, involving a dynamic interplay of genetic predispositions, structural and electrical remodeling of the atria, and systemic modulating factors [12, 13]. Key mechanisms include ectopic triggers, predominantly from the pulmonary veins (PVs), and an arrhythmogenic substrate characterized by atrial fibrosis, inflammation, oxidative stress, and heterogeneous electrical properties [14–16]. The “AF begets AF” phenomenon underscores the self-perpetuating nature of the arrhythmia, where repetitive episodes drive further atrial changes that facilitate its maintenance and progression [17–20]. Contemporary research is exploring the roles of advanced imaging, genomics, and multi-omics approaches in unraveling the underlying molecular and cellular pathways, promising more targeted therapeutic strategies [13, 21].

Current clinical guidelines, notably the 2023 ACC/AHA/ACCP/HRS and the 2020 ESC guidelines, advocate for an integrated, patient-centered approach to AF management [22, 23]. This comprehensive strategy emphasizes individualized stroke prevention, symptom control through rate or rhythm management, and aggressive modification of cardiovascular risk factors. Catheter ablation, especially pulmonary vein isolation (PVI), has emerged as a cornerstone and, in selected populations/trials, demonstrated superior efficacy over antiarrhythmic drugs in maintaining sinus rhythm and improving quality of life for symptomatic patients [24–26]. Indeed, recent meta-analyses further support catheter ablation as a first-line therapy in select populations [27]. However, despite these advancements, significant challenges persist, particularly for patients with persistent or long-standing persistent AF. Existing thermal ablation modalities carry inherent risks of collateral tissue damage to adjacent structures like the esophagus and phrenic nerve, limiting energy delivery and potentially impacting long-term lesion durability and procedural safety [28, 29].

These limitations have spurred the search for novel, safer, and potentially more effective ablation technologies. Recent innovations encompass diverse strategies including non-thermal energy modalities, advanced mapping and imaging platforms, hybrid procedural approaches, and upstream pharmacologic interventions targeting the arrhythmogenic substrate [30]. Among non-thermal modalities, pulsed-field ablation (PFA), employing irreversible electroporation, has garnered considerable attention due to its myocardium-selective tissue effect [31, 32]. By delivering ultra-rapid, high-voltage electrical fields, PFA achieves lesion formation while theoretically reducing collateral injury to adjacent non-cardiac structures, a feature that distinguishes it from conventional thermal methods. Concurrently, developments in high-resolution electroanatomic mapping, contact-force sensing catheters, and biomarker-guided upstream therapies are refining procedural precision and addressing the substrate-level drivers of AF recurrence. Collectively, these emerging technologies aim to enhance procedural safety, improve long-term efficacy, and enable more individualized treatment strategies, particularly for patients with complex or refractory AF.

This comprehensive review aims to provide an updated and in-depth overview of AF, tracing its evolution from its complex molecular and cellular pathogenesis to the latest diagnostic strategies and therapeutic interventions. While offering a balanced perspective on conventional treatments, this article critically evaluates the full spectrum of emerging technologies, including non-thermal ablation platforms, advanced mapping systems, and novel pharmacologic strategies, detailing their mechanistic foundations, clinical evidence, and translational potential. By synthesizing the most current data and addressing both the promise and the persisting challenges of these approaches, we seek to equip clinicians and researchers with actionable insights to navigate the evolving landscape of AF management and to advance more durable and personalized care, particularly for patients in whom traditional therapies have proven insufficient.

## Pathogenesis of AF

**Fig. 1:**
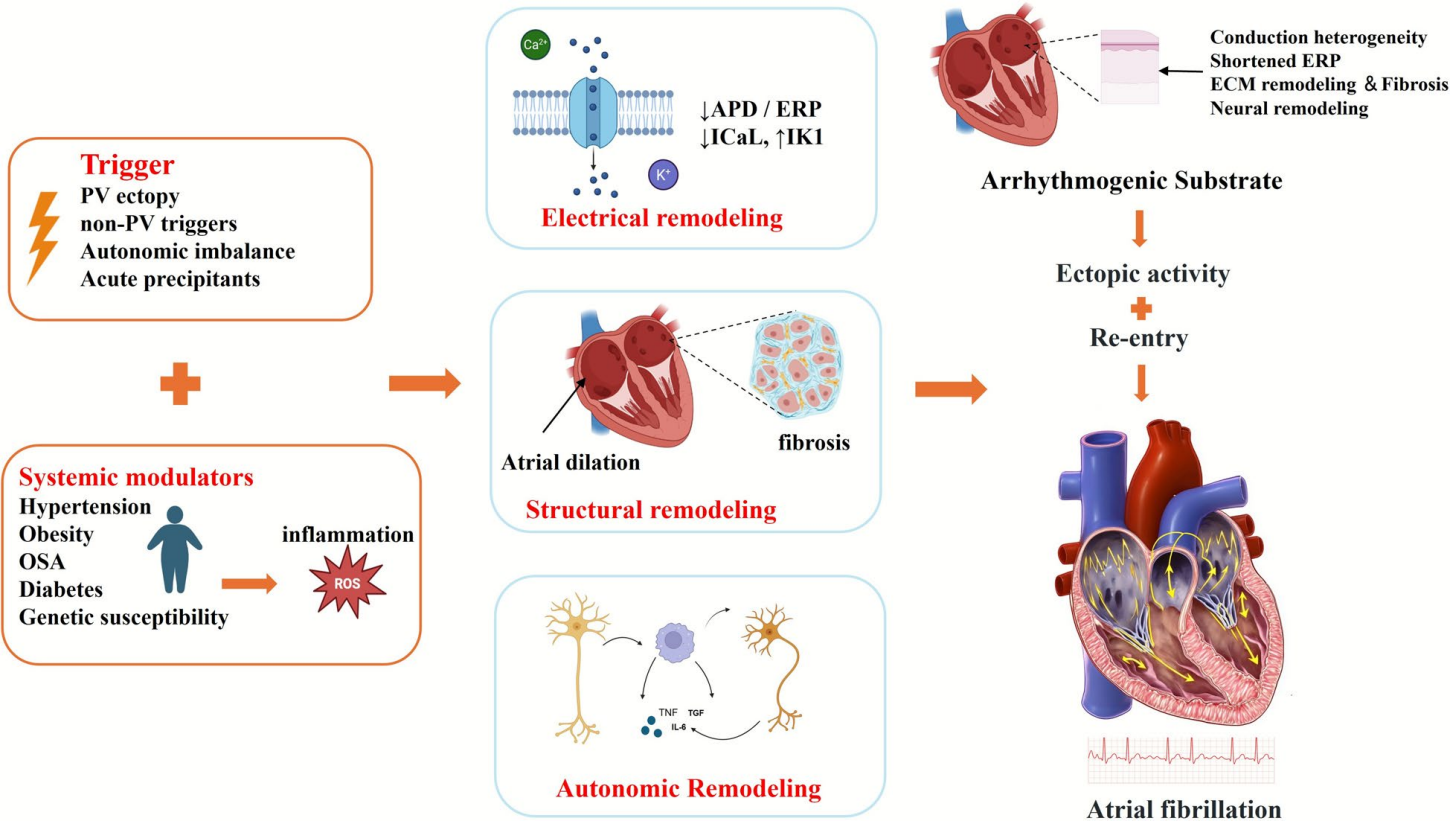
Pathogenesis of AF. Triggers such as pulmonary vein ectopy, non‑PV triggers, autonomic imbalance and acute precipitants together with systemic modulators including hypertension, obesity, obstructive sleep apnea, diabetes and genetic susceptibility drive three forms of atrial remodeling: electrical remodeling, structural remodeling and autonomic remodeling. These remodeling processes converge to form an arrhythmogenic substrate characterized by conduction heterogeneity, shortened effective refractory period, fibrosis and extracellular matrix remodeling, and neural remodeling. The substrate promotes ectopic activity and re‑entry, which produce sustained AF. AF in turn accelerates remodeling, creating a self‑sustaining feedback loop summarized by “AF begets AF.” Figure created with BioRender.com

The genesis and maintenance of AF reflect a dynamic interplay between initiating triggers, a progressively remodeled arrhythmogenic substrate, and systemic modulators that together create a self-sustaining arrhythmic milieu (Fig. 1). A clinically useful framework is to view AF as focal electrical impulses acting on an increasingly permissive atrial tissue environment; this explains why early AF is often trigger-dependent while advanced AF requires substrate-directed strategies. Recognizing the relative contribution of triggers, substrate and systemic factors in each patient is essential for diagnostic phenotyping and therapeutic selection [1, 14, 33–35]. Conceptually, AF can be embedded within the spectrum of “atrial cardiomyopathy,” wherein electrical, structural, metabolic, and autonomic alterations accumulate over time; early phases are dominated by trigger susceptibility, whereas later phases are driven by substrate complexity and systemic disease burden. Importantly, remodeling evolves on distinct time scales, electrical minutes to hours, autonomic hours to days, and structural weeks to months, providing a rationale for stage-specific interventions and longitudinal monitoring.

### Initiating triggers

Pulmonary veins remain the predominant source of initiating triggers, particularly in paroxysmal AF, because myocardial sleeves extending into the veins display distinct electrophysiological properties, shorter action potential duration, enhanced automaticity, and dense autonomic innervation that predispose to rapid focal discharges and afterdepolarizations [14, 36–39]. Autonomic fluctuations, showing abrupt sympathetic or vagal shifts, modulate myocardial excitability and calcium handling, increasing the likelihood of both early and delayed afterdepolarizations that can precipitate AF. Clinically important non-pulmonary venous triggers, for example, superior vena cava, crista terminalis, ligament of Marshall, coronary sinus, interatrial septum, and the left atrial appendage, share structural and electrophysiological features, myocardial sleeves, complex fiber orientation and rich autonomic input, that enable focal firing or localized re-entry and often become prominent in persistent or recurrent AF after PVI [40–43]. Accurate identification and targeted ablation of non-PV sources are critical when empirical PVI fails or when clinical presentation suggests atypical triggers [44–46].

At the cellular level, PV trigger formation is tightly linked to calcium cycling instability: RyR2 leak and CaMKII hyperactivation foster delayed afterdepolarizations under sympathetic drive, while action potential triangulation and dispersion under vagal predominance can promote early afterdepolarizations; adenosine-sensitive currents further shorten refractory periods in PV sleeves, enhancing ectopy. Acute precipitants, binge alcohol (“holiday heart”), acute infection/fever, intense endurance exercise, gastroesophageal reflux with vagal activation, and episodic obstructive sleep apnoea, transiently lower the threshold for ectopy via combined autonomic and mechano-electric effects, explaining the clustering of symptomatic paroxysms in susceptible individuals. Non-PV triggers frequently exhibit microreentry anchored in anisotropic fiber bundles or at myocardial–venous junctions; careful induction protocols and sequential mapping of the SVC, CS, septum, and LAA increase diagnostic yield, while recognizing that post-PVI substrate changes may unmask previously silent non-PV sources.

### Sustaining substrate and systemic modulators

Sustained AF depends on an atrial substrate shaped by interrelated electrical, structural and autonomic remodeling processes that create conduction heterogeneity, promote wavebreak and facilitate re-entrant circuits. Electrical remodeling typically manifests as shortening of action potential duration and effective refractory period, largely driven by altered ion channel expression, reduced L-type calcium current and increased inward rectifier potassium current, and disturbed intracellular calcium handling with increased sarcoplasmic reticulum leak and impaired reuptake; these changes favor both re-entry and triggered activity. Alterations in gap junctional proteins, notably connexins 40 and 43, impair cell-to-cell coupling and increase conduction anisotropy, heightening susceptibility to functional block [21, 47–49]. Structural remodeling is dominated by atrial fibrosis: collagen deposition creates anatomical barriers and low-voltage zones that fragment conduction and anchor re-entrant circuits; concomitant myocyte hypertrophy, apoptosis and fatty infiltration further disrupt conduction and mechano-electrical signaling [50–52]. Autonomic remodeling, regional changes in sympathetic and parasympathetic innervation and ganglionated plexi, both augments trigger formation and modulates substrate vulnerability, making autonomic modulation a potential adjunctive target [38, 53].

Beyond canonical ion channel shifts, constitutive activation of the acetylcholine-gated potassium current (I_K,ACh) and upregulation of I_K1 stabilize reentry by abbreviating atrial refractoriness, while subtle Na_v1.5 changes and reduced sodium current slow conduction, expanding the excitable gap. Mechano-electric feedback via stretch-activated channels (e.g., TRPC and Piezo) links atrial pressure/volume overload to acute conduction dispersion, providing a mechanistic bridge between haemodynamic stress and AF maintenance. Fibrogenic signalling is driven by RAAS activation, TGF-β/Smad pathway upregulation, and fibroblast–myocyte crosstalk; oxidative stress from NADPH oxidases (NOX2/4), mitochondrial dysfunction, and uncoupled eNOS amplifies matrix deposition and perturbs calcium homeostasis, creating a pro-arrhythmic loop. Epicardial adipose tissue (EAT) exerts paracrine effects (adipokines, inflammatory mediators) and can infiltrate the atrial myocardium, especially along the posterior wall, magnifying conduction heterogeneity. Autonomic remodeling is regionally heterogeneous: clusters of ganglionated plexi on the posterior left atrium and around the PVs facilitate rapid oscillations in refractoriness; heightened vagal tone shortens action potentials through I_K,ACh, while sympathetic surges increase calcium loading, together fostering spatially discordant alternans and wavebreak.

Systemic comorbidities, inflammation, oxidative stress and genetic predisposition substantially modulate substrate evolution and the clinical course of AF. Hypertension, heart failure, obstructive sleep apnoea, obesity and diabetes accelerate remodeling through atrial stretch, chronic inflammation, oxidative injury and pro-fibrotic signaling; thus, aggressive risk-factor management is integral to prevention and to improving outcomes after ablation [16, 54, 55]. Pro-inflammatory cytokines and reactive oxygen species directly alter ion channel function, activate fibroblasts and stimulate collagen deposition, creating a feed-forward loop between inflammation and fibrosis [16, 50]. Genetic and epigenetic factors identified by genome-wide studies and noncoding RNA research modulate individual susceptibility and treatment response, contributing to the marked heterogeneity observed in AF pathophysiology and clinical trajectories [21, 56]. Integrating clinical, molecular and imaging markers therefore enables more precise risk stratification and individualized therapy.

Additional systemic contributors include thyroid dysfunction, chronic kidney disease, and alcohol use, each accelerating atrial myopathy via distinct pathways (thyroid hormone–mediated adrenergic sensitization, uraemic inflammation, acetaldehyde-related oxidative injury). Intermittent hypoxia in OSA augments sympathetic tone and oxidative stress, while metabolic syndrome promotes lipotoxicity and EAT expansion. Genetic architecture extends beyond ion channel variants; developmental genes (e.g., PITX2) influence PV sleeve maturation and autonomic patterning, whereas noncoding RNAs (e.g., miR-21, miR-29) regulate fibroblast activation and matrix turnover. Age-related amyloid deposition and microvascular rarefaction further degrade atrial compliance and conduction, underscoring that “AF begets AF” through convergent molecular and tissue-level mechanisms.

### Translational implications: imaging, mapping and therapeutic stratification

Translating mechanistic insights into clinical practice requires high-resolution imaging and electrophysiological mapping to delineate the dominant arrhythmogenic mechanisms in each patient. CMR with late gadolinium enhancement quantifies atrial fibrosis and can identify substrate targets for modification, whereas high-density electroanatomic mapping detects low-voltage areas, fractionated electrograms and conduction block lines that may guide ablation beyond empirical PVI [52, 57]. Mapping techniques aimed at locating organized drivers or rotors offer a mechanistic basis for focused ablation but require further validation regarding incremental clinical benefit [58]. Intracardiac echocardiography(ICE) enhances anatomic visualization and catheter-tissue contact assessment, improving procedural safety. By integrating clinical risk factors, molecular signatures and multimodal imaging/mapping data, clinicians can move beyond a one-size-fits-all approach toward tailored interventions, choosing trigger-focused ablation in early, trigger-dominant disease and substrate-modifying strategies in advanced or persistent AF to optimize outcomes and reduce recurrence.

Standardized CMR workflows, including T1 mapping and quantification of late gadolinium enhancement (LGE) burden, enable staging of atrial fibrosis, which correlates with procedural success and thromboembolic risk; quantifying EAT volume and distribution adds complementary information on paracrine inflammatory load. Pre-procedural CT enhances anatomical segmentation for lesion planning and may predict challenging anatomies (common PV trunks, thick LAA ridge). In the EP lab, careful attention to voltage thresholds, rhythm state (sinus versus AF), and activation mapping density is essential to avoid overestimating low-voltage zones; adjunctive techniques (noninvasive ECGI, ripple/charge-density mapping) can identify localized drivers but should be applied with protocolized validation to mitigate false positives. Mechanism-guided stratification favors: (1) trigger-centric ablation (PVI ± targeted non-PV foci) in early, low-fibrosis phenotypes; (2) substrate modification (low-voltage area debulking, posterior wall isolation, linear lesions with rigorous bidirectional block) in advanced fibrosis; and (3) autonomic modulation (ganglionated plexus ablation, limited LOM ethanol infusion in select cases) when autonomic dominance is evident. Crucially, contemporaneous risk-factor optimization (weight loss, BP/OSA control, glycaemic management, alcohol moderation) is not ancillary but foundational, as it attenuates ongoing substrate progression and improves long-term freedom from AF, underlying the principle that effective preventive strategies are always essential for cardiovascular health [59]. A practical clinical pathway integrates serial phenotyping, combining symptoms, burden metrics from wearables, biomarker panels (e.g., natriuretic peptides, inflammatory markers), and imaging, with adaptive therapy selection, acknowledging that the arrhythmogenic phenotype can shift over time and warrants re-evaluation after index interventions.

## Diagnosis, classification and risk stratification

The comprehensive assessment of AF extends beyond mere arrhythmia identification, encompassing a structured approach to classify AF, evaluate thromboembolic risk, characterize its burden and underlying substrate, and ultimately guide individualized therapy. Contemporary guidelines emphasize a holistic, patient-centered evaluation, often summarized by the ‘4S-AF’ scheme, which covers stroke risk, symptom severity, AF burden and substrate severity [22, 23].

The initial suspicion of AF arises from diverse clinical presentations, ranging from asymptomatic detection during routine checks to severe symptoms such as palpitations, dyspnea, fatigue, or even initial presentation with stroke or heart failure exacerbation. A thorough medical history, including symptom characterization, comorbidities, and family history, is crucial [22, 23]. While physical examination may reveal an irregularly irregular pulse, definitive diagnosis relies on electrocardiographic evidence.

The electrocardiogram (ECG) remains the cornerstone for AF diagnosis, with diagnosis requiring a recording of at least 30 s on a standard 12‑lead ECG or single‑lead tracing that shows an irregularly irregular RR interval and absence of discrete P waves, replaced by fibrillatory waves [22, 23]. For paroxysmal AF, given its intermittent nature, prolonged ECG monitoring is indispensable. Advancements in cardiac monitoring technologies have significantly enhanced detection rates: from conventional 24-h Holter monitors to extended patch recorders and implantable cardiac monitors (ICMs) for long-term surveillance [60]. Wearable devices that use photoplethysmography (PPG) provide useful opportunistic screening; studies such as the Apple Heart Study demonstrated the feasibility of smartwatch‑detected irregular pulses, although such findings require confirmatory ECG or clinician review [61]. For patients with cryptogenic stroke, ICMs have proven superior to conventional monitoring in revealing underlying AF [62].

Classification of AF based on its temporal pattern guides prognosis and management: Paroxysmal AF self-terminates spontaneously or with intervention within 7 days; Persistent AF lasts longer than 7 days or requires cardioversion for termination; Long-standing Persistent AF is continuous AF lasting over 1 year where a rhythm control strategy is pursued; and Permanent AF signifies an accepted form of AF where rhythm control is no longer pursued by mutual patient-physician decision.

Central to AF management is the meticulous assessment of thromboembolic and bleeding risks. The CHA2DS2-VASc score is the guideline‑recommended tool for stratifying ischemic stroke risk, guiding the initiation of oral anticoagulation (OAC). This score considers factors such as congestive heart failure, hypertension, age, diabetes mellitus, prior stroke/TIA/thromboembolism, vascular disease, and sex category. Current guideline thresholds for OAC should be followed, for example, many guidelines recommend OAC for men with CHA2DS2‑VASc ≥ 2 and for women with CHA2DS2‑VASc ≥ 3, but practitioners must consult the most recent guidance and individualize decisions. Conversely, the HAS-BLED score is commonly used to assess bleeding risk, aiding in shared decision-making regarding OAC initiation and ongoing management. For patients at low stroke risk, OAC may not be indicated, while for higher-risk patients, OAC is strongly recommended.

Imaging plays a pivotal role in characterizing atrial substrate and assessing for cardiac structural abnormalities and thrombus. Echocardiography, both transthoracic (TTE) and transesophageal (TEE), is fundamental. TTE assesses left atrial size and function, for example, left atrial volume index and left atrial strain by speckle‑tracking, ventricular function and valvular heart disease [63, 64]. TEE remains the reference standard for excluding LAA thrombus, particularly before cardioversion or catheter ablation. Cardiac magnetic resonance (CMR) offers high-resolution visualization of atrial anatomy and, crucially, can quantify atrial fibrosis using LGE, providing substrate information that may predict AF progression and ablation outcomes, although LGE quantification in the thin‑walled atrium has technical limitations and interstudy variability [50, 51]. Cardiac computed tomography (CT) is primarily utilized for detailed anatomical mapping of the LA and PVs prior to ablation procedures, and can also offer insights into LAA morphology.

Beyond routine tests, biomarkers are emerging as adjuncts for risk stratification. Elevated natriuretic peptides (NT-proBNP) and high-sensitivity cardiac troponins (hs-cTn) reflect atrial stretch/pressure and myocardial injury, respectively, correlating with adverse outcomes in AF [65–68]. Novel markers, such as growth differentiation factor‑15 (GDF‑15) and selected microRNAs, show promise for predicting AF recurrence after cardioversion or ablation and for identifying patients at higher risk of progression, potentially guiding the intensity or choice of rhythm‑control strategies [69–73]. For instance, a higher NT-proBNP may indicate a more advanced substrate, potentially influencing the decision towards more aggressive rhythm control or a more complex ablation strategy [74–76]. However, these biomarkers require further validation before routine incorporation into widespread clinical decision algorithms.

In conclusion, diagnosis and comprehensive characterization of AF require an integrated approach that begins with clinical assessment and ECG confirmation, proceeds with precise classification and robust stroke/bleeding risk evaluation, and incorporates advanced imaging for substrate characterization. The 4S-AF framework supports individualized decisions on anticoagulation, rate versus rhythm control and candidacy for catheter ablation, with the ultimate goals of optimizing clinical outcomes and improving quality of life. Ongoing research into artificial-intelligence-driven diagnostics and novel biomarkers promises to refine patient selection and therapeutic personalization further.

## Traditional therapies

The comprehensive management of AF has undergone a significant evolution, shifting from a narrow focus on isolated rhythm or rate control to an integrated, patient-centered approach. This contemporary paradigm, often encapsulated by the “ABC” pathway, emphasizes the crucial need to address stroke prevention, symptom relief, and underlying risk factors in a coordinated manner [22, 23]. Within this framework, traditional therapeutic modalities, encompassing pharmacological interventions for rate and rhythm control, electrical cardioversion, and established catheter ablation techniques, form the foundational pillars of AF care [77, 78].

### Rate control strategies

Rate control aims to alleviate symptoms and prevent tachycardia-induced cardiomyopathy by maintaining an acceptable ventricular response rate during AF. Current guidelines generally endorse a lenient resting target of < 110 bpm for asymptomatic or minimally symptomatic patients, while recommending a stricter target (≈ < 80 bpm at rest) for highly symptomatic patients or those with left ventricular dysfunction. This strategy is often preferred for elderly patients, those with minimal symptoms, or individuals for whom rhythm control attempts have proven unsuccessful or are contraindicated due to comorbidities or patient preference. First-line pharmacological agents include beta-blockers, non-dihydropyridine calcium channel blockers, and digoxin. Beta-blockers are frequently favored due to their additional benefits in patients with concomitant coronary artery disease or heart failure with reduced ejection fraction (HFrEF) [22, 23, 79–81]. Calcium channel blockers are effective but require careful consideration in HFrEF. Digoxin, while effective for resting rate control, has a slower onset of action and limited efficacy during exertion. The choice of agent is highly individualized, taking into account comorbidities, potential side effect profiles, and patient tolerance. While effective in mitigating symptoms and preventing rate-related complications, rate control does not address the underlying arrhythmia or its long-term sequelae such as atrial remodeling, and a significant proportion of patients may remain symptomatic despite adequate rate management [57].

### Rhythm control strategies: antiarrhythmic drugs and cardioversion

Rhythm control aims to restore and maintain sinus rhythm, thereby improving symptoms and potentially preventing adverse outcomes associated with AF, such as stroke, heart failure progression, and cognitive decline, particularly in younger, symptomatic patients or those with AF-related cardiomyopathy.

#### Antiarrhythmic Drugs (AADs)

AADs represent the initial pharmacological approach for rhythm control. These agents are classified based on their primary effects on cardiac ion channels (Vaughan Williams classification). Class Ic drugs (e.g., flecainide, propafenone), potent sodium channel blockers, are suitable for patients without significant structural heart disease. Class III agents, like amiodarone, sotalol, dofetilide and dronedarone, primarily block potassium channels, thereby prolonging repolarization. Amiodarone is highly effective across various AF types but is associated with a cumulative burden of significant extracardiac toxicities, like pulmonary fibrosis, thyroid dysfunctionand and hepatic damage, with long-term use, limiting its first-line utility in many patients. Sotalol, possessing both beta-blocking and potassium channel-blocking properties, necessitates careful monitoring for QT prolongation and proarrhythmia. Dronedarone offers a safer profile than amiodarone but is contraindicated in symptomatic heart failure and permanent AF [82, 83]. Dofetilide is effective, particularly in heart failure patients, but requires inpatient initiation and careful dose titration.

Despite their utility, AADs demonstrate modest efficacy in maintaining sinus rhythm, with success rates typically ranging from 30–60% at one year. More critically, their long-term effectiveness is often suboptimal, and they are associated with a considerable burden of extracardiac toxicities and proarrhythmic risks [24, 84, 85]. These significant limitations frequently lead to poor patient adherence and restrict their long-term clinical utility, particularly in persistent AF where efficacy is even lower due to more extensive atrial remodeling, thereby underscoring the urgent need for more effective and safer alternative strategies.

#### Electrical cardioversion

For acute restoration of sinus rhythm, particularly in hemodynamically unstable patients or those with severe symptoms, electrical cardioversion is the most rapid and effective method, delivering a synchronized electrical shock to depolarize a critical mass of myocardium and reset the heart’s rhythm. Pharmacological cardioversion using intravenous AADs, like flecainide, propafenone for patients without structural heart disease; amiodarone, ibutilide for sustained AF, can also be effective, especially for recent-onset AF. Pre-procedure anticoagulation is critical to prevent thromboembolic events, especially if AF duration is unknown or exceeds 48 h. While highly effective acutely, cardioversion does not prevent AF recurrence, and maintaining sinus rhythm often requires subsequent AAD therapy or catheter ablation.

### Catheter ablation: a cornerstone of rhythm control

Catheter ablation has emerged as a cornerstone rhythm control strategy for symptomatic AF refractory to AADs, and increasingly as a first-line therapy in selected patients, consistently demonstrating superior efficacy in maintaining sinus rhythm and improving quality of life compared to AADs [84–89]. The fundamental principle involves creating targeted lesions within the atria to electrically isolate arrhythmogenic triggers and/or modify the pro-arrhythmic substrate.

#### Radiofrequency (RF) ablation

RF ablation is the most established catheter-based technique, utilizing high-frequency alternating current to generate resistive heating in myocardial tissue. Temperatures typically reach 50–70 °C, inducing irreversible coagulative necrosis and forming discrete, permanent lesions that block abnormal electrical conduction [29, 90]. RF ablation, while highly versatile and adaptable to diverse anatomical complexities, typically involves a longer learning curve to achieve consistent transmural lesions and minimize complications, often requiring 50–100 cases for an operator to reach proficiency in complex AF ablation procedures.

The cornerstone of RF ablation for AF is PVI, based on the seminal observation that ectopic beats originating predominantly from the PV sleeves serve as critical triggers for AF [36]. The goal is to electrically isolate all four PVs from the left atrium, preventing these triggers from propagating into the atrial myocardium. This is typically achieved by creating continuous circumferential lesions around the PV ostia or antra. PVI alone is highly effective for paroxysmal AF, with one-year success rates ranging from 70–85% in experienced centers [91–93]. However, for persistent AF, the efficacy of PVI alone is lower, often necessitating additional ablation targets due to more complex and widespread atrial remodeling [47].

To improve outcomes in persistent AF, various adjunctive RF ablation strategies have been explored beyond PVI. These include creating linear lesions, like roof and mitral isthmus lines, to compartmentalize the atrium and interrupt macro-reentrant circuits, and ablating complex fractionated atrial electrograms (CFAEs), thought to represent areas of fibrillatory drivers or fibrotic substrate. However, the incremental benefit of these extensive lesion sets over PVI alone for persistent AF remains contentious, with landmark trials showing no significant added advantage of CFAE ablation [51, 94–99]. The challenge lies in reliably identifying truly critical non-PV targets that sustain persistent AF.

A significant technical refinement in RF ablation, High Power Short Duration (HPSD) involves delivering higher power (e.g., >  40 W) for shorter durations, typically 4–10 s, at each lesion site. This approach aims to achieve faster, more efficient, and transmural lesion formation by rapidly heating tissue while potentially reducing tissue edema and significantly lowering the risk of steam pop formation and charring [100]. Recent studies, including the Q-FFICIENCY trial, have demonstrated that HPSD protocols can significantly shorten procedure times, improve workflow efficiency, and are non-inferior in safety and efficacy compared to conventional RF settings, thereby enhancing patient throughput and potentially reducing procedural complications [101, 102]. Nevertheless, careful attention to contact force, impedance, and temperature monitoring remains crucial to avoid complications.

#### Cryoballoon Ablation (CBA)

CBA represents a distinct nonthermal approach to PVI, employing a balloon catheter filled with a refrigerant (e.g., nitrous oxide) to cool tissue rapidly to temperatures typically between −30 °C and −60 °C. This induces cell death via freezing and thawing cycles, forming continuous, transmural lesions [103–105]. CBA offers several operational advantages, including a simpler “single-shot” approach for PVI, which contributes to a shorter learning curve and potentially faster procedure times, especially for operators gaining initial experience [106]. Cryoballoon ablation, with its ‘single-shot’ approach for PVI, generally offers a steeper and shorter learning curve compared to point-by-point RF ablation, often reaching proficiency for PVI within 20–30 cases. The large, contiguous lesions created by the balloon are often transmural, and the cryoenergy itself may be less damaging to adjacent non-myocardial tissues [28]. Furthermore, the reversible nature of early cryoablation allows for a “test freeze” to assess phrenic nerve function, potentially mitigating the risk of permanent phrenic nerve palsy.

The FIRE AND ICE trial established CBA as non-inferior to RF ablation for paroxysmal AF regarding efficacy and safety, with comparable rates of AF recurrence [107, 108]. Subsequent studies, including the ESCAPE-AF trial, have explored CBA in persistent AF, demonstrating non-inferiority to RF ablation in terms of freedom from atrial tachyarrhythmias, albeit with a numerically higher complication rate in the Cryo group in some analyses [25]. While highly effective for PVI, CBA’s utility is primarily limited to PV isolation. Its “one-size-fits-all” balloon approach makes it less adaptable for ablating non-PV triggers or complex extrapulmonary substrate in persistent AF that require precise, point-by-point lesions [105]. Other considerations include the risk of phrenic nerve injury and potential for transient esophageal injury.

For paroxysmal AF, both RF and CBA are highly effective rhythm control strategies with comparable long-term efficacy and overall safety profiles, as evidenced by large randomized controlled trials and meta-analyses [104, 109, 110]. The choice between these modalities often depends on operator experience, anatomical considerations, institutional preferences, and the specific patient’s clinical profile. RF ablation offers greater versatility for complex anatomies and non-PV targets, while CBA may be favored for its speed and simplicity in pure PVI cases, particularly in less experienced centers. Both modalities demand a significant learning curve to optimize outcomes and minimize complications. While both modalities demand a significant learning curve to optimize outcomes and minimize complications, the learning curve for RF ablation for complex AF cases is generally longer than for cryoballoon PVI, reflecting the differing procedural complexities.

### Non-catheter ablation methods: surgical and hybrid approaches

For select patients, particularly those undergoing concomitant cardiac surgery, like valve repair or replacement, CABG, or those with advanced, highly symptomatic persistent AF refractory to conventional catheter ablation, surgical or hybrid ablation techniques may be considered.

#### Surgical Maze procedure

The original “cut-and-sew” Maze procedure involves creating a series of transmural incisions in the atria to interrupt all potential re-entrant pathways while preserving atrial contractile function [111–114]. Modified Maze procedures utilize various energy sources to create similar lesion patterns. Surgical ablation, particularly the Maze procedure, offers high success rates for rhythm control, especially when performed concomitantly with other cardiac surgeries, but is more invasive and associated with higher perioperative risks compared to catheter-based approaches [115–119].

#### Hybrid ablation

A hybrid approach combines epicardial ablation with endocardial catheter ablation. This allows for more extensive lesion creation, particularly on the posterior left atrium and targeting GPs, which can be challenging to access or ablate transmurally endocardially [120–124]. Hybrid procedures are typically reserved for patients with advanced, long-standing persistent AF or those with failed prior endocardial ablations, aiming to leverage the strengths of both approaches for improved outcomes [122, 125–127]. However, their complexity, resource intensity, and the need for multidisciplinary expertise limit widespread adoption.

In summary, traditional AF therapies offer a robust toolkit for managing this complex arrhythmia. While pharmacological approaches remain essential for rate and initial rhythm control, catheter ablation, particularly PVI, has transformed AF management for symptomatic patients, with ongoing refinements in RF and cryoballoon technologies. Surgical and hybrid approaches provide further options for challenging cases, underscoring the continuously evolving landscape of AF treatment.

## Novel treatment options

The limitations of conventional thermal ablation, non-selective collateral injury, variable lesion durability, and workflow inefficiencies, have accelerated the development of tissue-selective strategies for AF. Among these, PFA, which relies on IRE, has emerged as the most transformative concept. Unlike resistive heating or freezing, IRE produces non-thermal, myocardium-selective injury with preservation of the extracellular scaffold and microvasculature, a histologic signature that plausibly underpins the low rates of esophageal injury, phrenic palsy, and PV stenosis observed in clinical practice [128, 129].

### Mechanistic foundations: dose, selectivity, and lesion biology

#### IRE basics and myocardial selectivity

**Fig. 2:**
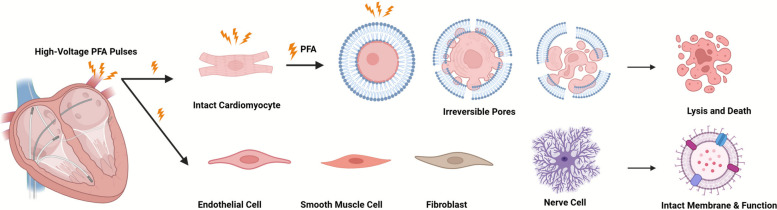
Mechanistic foundations of PFA and myocardial selectivity. High-voltage, microsecond-duration electrical pulses induce IRE in cardiomyocytes due to their inherently lower electroporation threshold. This leads to the formation of permanent nanopores, subsequent cell lysis, and selective ablation of myocardial tissue. Other cell types, like endothelial cells, smooth muscle cells, fibroblasts, nerve cells, possess higher electroporation thresholds, allowing them to resist irreversible electroporation and thus preserving the extracellular matrix and microvasculature. Figure created with BioRender.com

PFA delivers microsecond-duration pulses that generate electric fields on the order of several hundred to approximately 1,500 V/cm at the tissue interface. When the induced transmembrane potential surpasses a critical threshold (~ 0.5–1.0 V), lipid-bilayer nanopores form; if pore density and size exceed cellular repair capacity, irreversible electroporation ensues, culminating in cell death (Fig. 2). Cardiomyocytes, characterized by large surface area, high membrane capacitance, and a prominent transverse-tubule network, are preferentially susceptible. In contrast, fibroblasts, endothelium, and neural elements often require higher field strengths [30, 130–132]. Because IRE lacks thermal protein denaturation, the extracellular matrix (collagen, elastin) and microvasculature are largely preserved. This translates into sharply demarcated, homogeneous lesions without coagulum, steam pops, or carbonization that are hallmarks of thermal injury [29, 133–136]. Biphasic waveforms further modulate safety and tolerability by limiting net DC offset, mitigating pH shifts, and reducing skeletal muscle capture [132, 134].

#### Dose–response, sublethal zones, and anatomical heterogeneity

A defining feature of IRE is the steep field gradient surrounding the electrodes. Lesion cores, where the field exceeds the irreversible threshold are encircled by sublethal regions with near-threshold exposure [131, 132, 137]. In these border zones, transient nanopores may reseal or trigger delayed apoptosis rather than immediate necrosis. Live-cell and patch-clamp experiments reveal calcium influx, mitochondrial stress, and caspase activation over hours to days [138, 139]. In animal models, lesions mature into stable fibrosis, but interfaces of necrotic core and surviving myocardium can display dense collagen bands that alter conduction velocity and refractoriness [135, 140]. Early human late-gadolinium enhancement MRI suggests lesion stability across months without aneurysmal dilation [138, 141]. Clinically, minimizing sublethal rims, by ensuring adequate field strength across the full wall thickness and avoiding low‑dose “feathering” at the PV antra, likely reduces dormant conduction and late reconnection.

Anatomy and tissue biophysics modulate the delivered field. Atrial myocardial anisotropy favors current flow along fiber tracts, producing elongated lesions; transverse sheets and fibrosis can disrupt field propagation and create irregular edges [107]. High-flow blood pools may shunt current, and suboptimal electrode apposition at curved ostia or ridges creates “cold spots” below the IRE threshold [142, 143]. Finite-element models that incorporate anisotropic conductivity and realistic chamber geometry align with ex vivo and mapping-derived lesion patterns [144, 145]. These insights justify the clinical emphasis on: (1) R-wave–synchronized biphasic dosing to standardize delivery; (2) ICE-guided contact optimization to homogenize field distribution; and (3) anatomy-informed pulse sets, like increasing pulse counts or higher amplitude at thicker segments or carina [146].

#### Histopathologic validation and safety correlates

Large-animal studies and myocardial-slice models consistently demonstrate cardiomyocyte death with preservation of collagen/elastin scaffolds and microvasculature; ultrastructurally, membrane disruptions are evident without thermal coagulation artifacts [135, 147, 148]. Multimodality imaging (contrast-enhanced MRI, PET) supports transmurality and microvascular integrity over weeks to months [149]. This biology plausibly explains the markedly reduced risk of esophageal injury and permanent phrenic nerve palsy seen with PFA relative to thermal energy [150, 151]. It also underlies specific event profiles: absence of charring/steam pops, but potential for transient skeletal muscle capture and, rarely, hemolysis if intravascular exposure to intense fields occurs. Mechanism-anchored safety practice therefore centers on (1) synchronization and neuromuscular blockade to limit extracardiac capture; (2) catheter handling that avoids intravascular pulse delivery; and (3) vigilance for coronary vasospasm during posterior left atrial applications, treated promptly with intracoronary vasodilators.

### Standardized procedural workflow and learning curve

The clinical deployment of PFA should be standardized around outcome-relevant, platform-independent principles with the same therapeutic endpoint as thermal ablation in Sect. “Traditional therapies”: durable, contiguous antral PVI verified by rigorous testing. Three elements primarily determine lesion quality across systems: (1) dose sufficiency across the entire antrum and wall thickness; (2) uniform electric-field distribution through stable catheter geometry and verified tissue apposition; and (3) synchronized biphasic delivery to minimize extracardiac capture and enhance tolerability [23, 29]. Procedural success should be adjudicated with pragmatic, device-agnostic endpoints, first-pass isolation, bidirectional block, and pharmacologic provocation, facilitating cross-technology comparability [135, 151–153].

Preprocedural planning integrates established clinical and anatomic criteria (paroxysmal vs. persistent AF, left atrial size, comorbidities) with high-resolution imaging to delineate wall thickness, PV ostial dimensions, ridge/carina anatomy, and pre-existing scar that may influence transmurality and dosing strategy [23, 29]. Anticoagulation protocols should ensure uninterrupted coverage and early intraprocedural therapeutic anticoagulation; neuromuscular blockade and reliable R-wave synchronization minimize catheter motion and skeletal muscle capture. ICE is arranged not only for transseptal guidance but also for real-time assessment of catheter–tissue apposition and early recognition of atypical responses or microbubbles, given the absence of immediate thermal feedback with PFA [154–156].

**Fig. 3:**
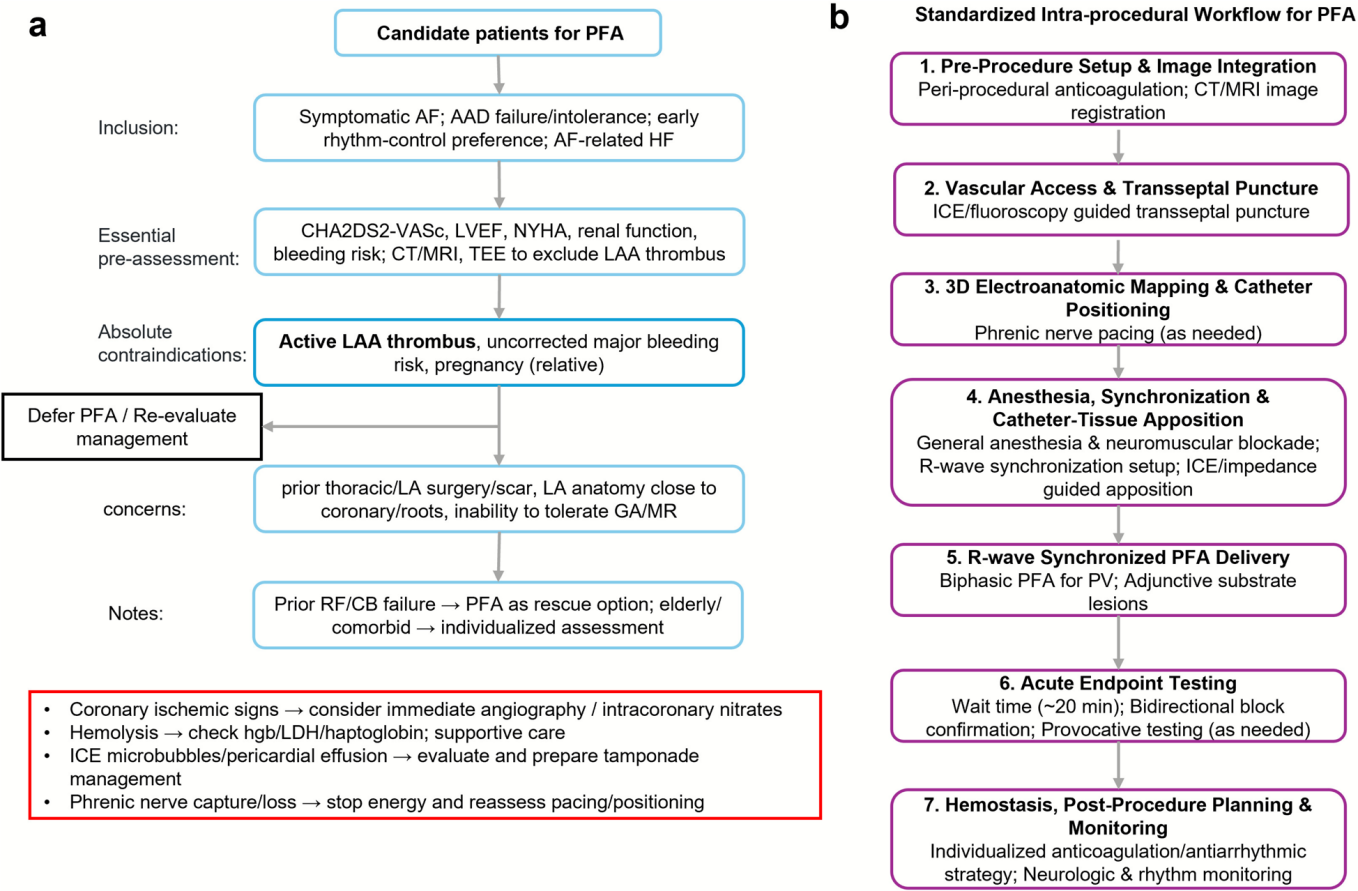
Standardized workflow and critical considerations for PFA in AF. This flowchart illustrates the standardized workflow for PFA in AF. **a** Outlines patient inclusion criteria, essential pre-assessment items, absolute contraindications, and other clinical concerns requiring individualized evaluation. A decision node addresses the presence of LAA thrombus or inadequate anticoagulation. **b** Depicts the seven key intra-procedural steps for PFA delivery, encompassing initial setup, vascular access and transseptal puncture, 3D electroanatomic mapping, anesthesia and catheter-tissue apposition verification, R-wave synchronized energy delivery, acute endpoint testing, and immediate post-procedure planning. Intra-procedural critical alerts & actions (red box): This section provides concise guidance for the immediate recognition and management of critical events that may occur during the PFA procedure

The PFA ablation sequence, adhering to a robust, standardized algorithm, is fundamentally distinct from thermal approaches. It typically encompasses seven key steps: (1) femoral venous access and transseptal puncture under fluoroscopic and ICE guidance; (2) comprehensive 3D electroanatomic mapping and precise registration of pre-acquired imaging datasets; (3) meticulous verification of electrode contact force and geometry, which are critical for achieving uniform electric field distribution and transmurality, especially in complex atrial anatomies and particularly important given the nonthermal nature of PFA, where immediate visual cues of lesion formation are absent. Mapping systems, when integrated with PFA catheters, provide real-time impedance monitoring, electrogram attenuation, and lesion tagging, which are important for confirming energy delivery and ensuring contiguous lesion creation; (4) R-wave–synchronized, biphasic pulse delivery for swift and circumferential pulmonary vein isolation, utilizing various catheter designs (basket, lattice-tip, or balloon systems); (5) judicious application of adjunctive linear lesions (e.g., posterior wall, roof line, mitral isthmus) as indicated by detailed substrate characterization, particularly in persistent AF where PVI alone may be insufficient; (6) acute efficacy testing with bidirectional block using advanced mapping techniques, often complemented by adenosine or isoproterenol challenges to unmask dormant conduction and identify critical gaps – a particular concern with non-thermal lesions that lack immediate visual cues; and (7) meticulous hemostasis and comprehensive postprocedural monitoring [135]. The implementation of a standardized flowchart (Fig. 3) is vital for enhancing team coordination and reducing procedural variability, while predefined “troubleshooting” protocols (e.g., low-voltage test pulses at anatomically challenging sites, readiness for intracoronary nitroglycerin) are crucial for the prompt and safe management of uncommon events such as coronary spasm or transient hemolysis, reflecting the unique safety considerations of electrical ablation [157–159].

PFA exhibits a distinct but relatively short learning curve. Proficiency typically stabilizes after approximately 30–50 cases, at which point first-pass isolation rates routinely exceed 90%, acute PVI can be achieved within left atrial dwell times compatible with streamlined workflows, and major complications remain below 1%, aligning with contemporary benchmarks [147, 151–153]. Compared with conventional point-by-point radiofrequency ablation, the curve is shorter; relative to cryoballoon ablation, it is at least comparable when accounting for balloon sizing and phrenic nerve management.

This learning curve appears to be notably steeper and shorter compared to the more protracted learning phase typically observed with conventional point-by-point radiofrequency ablation and at least comparable to, if not slightly faster than, cryoballoon ablation when considering the nuances of balloon sizing and phrenic nerve management. To accelerate this learning curve and ensure safe, rapid adoption, a structured and comprehensive training curriculum is paramount. This should include hands-on workshops with animal models, virtual-reality simulators for procedural familiarity, proctored live cases for real-world experience, and periodic peer-review of procedural metrics to foster continuous improvement [29]. Training protocols for PFA, while sharing commonalities with thermal ablation, place unique emphasis on understanding IRE biophysics, mastering R-wave synchronization, and recognizing unique PFA-specific safety considerations (e.g., potential for hemolysis, coronary spasm, and skeletal muscle stimulation) [157, 158]. Proctored cases focus on optimizing pulse delivery parameters and real-time ICE interpretation for contact assessment, which are critical in the absence of immediate thermal feedback on lesion formation. Programs should track platform-agnostic quality indicators—first-pass isolation, touch-up rates, procedure and fluoroscopy time, left atrial dwell time, and standardized safety endpoints to support operator credentialing and continuous quality improvement [147, 152, 153]. This outcome-focused, anatomy-informed, and reproducible approach is central to preserving PFA’s efficiency and safety advantages while ensuring durable clinical results.

### Clinical efficacy, durability and safety

#### Key randomized trials and multicenter registries

Three landmark randomized controlled trials have defined PFA’s acute efficacy and safety profile in AF ablation (Table 1). The ADVENT trial [138] (n≈350 paroxysmal AF) established PFA’s non-inferiority to thermal ablation for 12-month arrhythmia-free survival (82% vs. 80%), while demonstrating superior acute PVI (100% vs. 97%, *p* = 0.03) and a clinically significant halving of major complications (0.8% vs. 4.2%) [138, 160, 161]. For more complex substrates, the SPHERE Per-AF trial [162] (*n* = 250 persistent AF) demonstrated PFA’s superiority over point-by-point RF, with 12-month arrhythmia freedom of 73.8% versus 65.8% (*p* = 0.04). Critically, this trial reported zero esophageal injuries in the PFA cohort compared to 5% in the RF arm, underscoring a substantial safety advantage [162, 163]. The SINGLE-SHOT CHAMPION trial [138] (*n* = 200 paroxysmal AF), established balloon-based PFA as non-inferior to cryoballoon ablation for one-year arrhythmia freedom (78% vs. 76%, *p* = 0.67), alongside operational gains including a 30% reduction in procedure time and an 80% reduction in phrenic nerve palsy.

**Table 1:** Key randomized trials and registries in PFA for AF

Study (Design, n)	Population	PFA Protocol	Comparator	Acute PVI (%)	12-mo AF-free (%)	Major Complication (%)
ADVENT (RCT, ≈350) [138]	Paroxysmal AF	Farawave 1 800 V/100 µs × 5–10	RF/Cryo	100 vs 97^1^	82 vs 80	0.8 vs 4.2^2^
SPHERE Per-AF (RCT, 250) [163]	Persistent AF	Sphere-9 2 200 V/50 µs × 3–6	Point-by-point RF	—	73.8 vs 65.8^3^	1.4 vs 1.0^4^
SINGLE-SHOT CHAMPION (RCT, 200) [138]	Paroxysmal AF	Volt-AF 1 000–1 500 V/100–200 µs × 10–12	Cryoballoon	—	78 vs 76	—
Reddy et al. (2021) (Pooled FST, 121) [164]	Paroxysmal AF	Farawave (optimized biphasic)	None	100	84.5 (PFA-OW)	2.5 (CEC)/3.3
Osmancik P et al. (2024) (RCT) [158]	AF	PFA vs RF (Farapulse)	Radiofrequency Ablation	—	—	—
MANIFEST-17 K (Registry, 17,642) [165]	Paroxysmal (57.8%)/Persistent (35.2%) AF	Farawave (pentaspline, 1.8–2.0 kV)	None	N/A	N/A	0.98
PULSED-AF (Registry, 1 068) [151]	All comers	Mixed platforms	—	99.5	85	0.5
ADMiRE (Registry, 850) [153]	Paroxysmal AF	inspIRE	—	100	88	0.6
MANIFEST-REDO (Registry, 427) [165]	Redo AF	Mixed platforms	—	97	75 (opt. dosing)	2.8
EU-PORIA (Registry, 1 233) [147]	All comers	Farawave	—	> 99	82.6 (redo)	0.8

^1^*p* = 0.03 for acute PVI

^2^*p* < 0.01 for major complications

^3^*p* = 0.04 for AF-free survival

^4^Non-inferiority margin 8%

These randomized trial data are corroborated by large-scale, real-world registries that reflect broader patient demographics and operator experience. The PULSED-AF registry [151] (*n* = 1 068) reported 99.5% acute PVI and 85% one-year arrhythmia freedom. And ADMiRE [153] (*n* = 850) observed 100% acute isolation, 88% one-year success, and a remarkably low 0.6% major adverse event rate. However, it is imperative to acknowledge that these substantial initial outcomes, predominantly focusing on 6–12 month follow-up, are still predominantly short-to-medium term. A critical and overarching limitation across these foundational studies is the conspicuous absence of robust long-term (i.e., 2–5 year or greater) outcome data, which is absolutely essential for fully evaluating the sustained efficacy, durability, and comparative safety profile of PFA against established thermal modalities. This significant gap necessitates ongoing, well-powered clinical trials with extended follow-up periods to definitively assess PFA’s true long-term impact.

#### Acute efficacy and the challenge of lesion durability

PFA consistently delivers excellent acute procedural outcomes, with acute PVI rates exceeding 98% and first-pass isolation rates above 93% per vein across various catheter types in both paroxysmal and persistent AF cohorts [8, 138, 151–153, 163]. This high initial efficacy, however, is tempered by the challenge of long-term lesion durability, as PV reconnection remains the principal mechanism underlying late arrhythmia recurrence, a paradigm established with thermal ablation modalities. Twelve-month arrhythmia-free survival typically ranges from 82–88% in paroxysmal AF to 68–74% in persistent AF [8, 153, 154, 157]. While preliminary two-year data suggest sustained efficacy in the range of 75–80% [166], these observations are not yet substantiated by large-scale, long-term randomized trials.

Mechanistic insights into lesion non-durability have been advanced through dedicated redo procedure studies. The MANIFEST-REDO study identified that reconnections cluster at specific anatomical “cold spots”, notably the left superior/inferior veins and the carina, where complex geometry and variable tissue contact can lead to electric-field attenuation [165]. This spatial pattern suggests that field inhomogeneity, rather than a global energy deficit, is a primary determinant of lesion failure. Supporting this, the PULSE-EU study established a clear dose–response relationship, demonstrating that optimized protocols (≥ 1,800 V, ≥ 10 pulses/application) reduced three-month reconnection rates to below 15% [147]. At the tissue level, these clinical findings are corroborated by electrophysiological and histological evidence: residual bipolar electrogram voltage > 0.2 mV post-PFA reliably predicts reconnection, and histopathology confirms incomplete transmurality at these sites [167]. Collectively, these data underscore a central theme in PFA: despite its intrinsic tissue selectivity, the steep electric field gradients inherent to irreversible electroporation demand meticulous procedural technique and optimized energy delivery to consistently achieve transmural, and therefore durable, lesions across a heterogeneous atrial substrate.

Multivariate analyses have identified a consistent prognostic signature for one-year success, with favorable factors relating to either substrate complexity (paroxysmal AF, smaller left atrial volume) or the quality of energy delivery (optimized dosing, ICE-verified apposition, and operator experience > 30 cases) [168]. A significant hurdle to optimizing outcomes is the current lack of cross-platform dosing standardization, which complicates the interpretation of comparative trial data and impedes the development of universal best-practice protocols to maximize long-term arrhythmia freedom.

#### Patient-centered outcomes and impact on hard clinical endpoints

Beyond the primary endpoint of arrhythmia recurrence, emerging evidence suggests that PFA confers meaningful benefits on hard cardiovascular outcomes and health-related quality of life—domains of paramount importance for clinical decision-making. A cost-effectiveness analysis from the landmark ADVENT trial cohort revealed that PFA-treated patients experienced significantly fewer strokes or transient ischemic attacks (0.4% vs. 1.2%, *p* = 0.04) and a lower incidence of heart failure hospitalizations (3.1% vs. 5.8%, *p* = 0.02) at 12 months compared to those undergoing thermal ablation [165, 169]. In parallel, the inspIRE multicenter substudy demonstrated greater and more sustained improvements in disease-specific quality of life, as measured by the AFEQT score, at both one and two years. These patient-reported benefits were accompanied by objective reductions in rehospitalizations and less need for antiarrhythmic drug escalation, suggesting a durable clinical advantage [170, 171]. While these findings are hypothesis-generating and require confirmation in prospectively designed trials, they provide compelling initial evidence that PFA’s procedural advantages may translate into improved long-term cardiovascular health.

Comprehensive subgroup analyses further support PFA’s broad applicability across diverse and often challenging patient populations. PFA has demonstrated superior efficacy in elderly patients (≥ 75 years) without an increase in procedural complications, a key consideration in this vulnerable cohort [172]. It has also been associated with reduced cardiovascular hospitalizations in patients with a high burden of comorbidities [173, 174] and has maintained efficacy even in the presence of significant left atrial enlargement. In the particularly challenging setting of redo procedures after prior thermal ablation failure, optimized PFA dosing achieved a 75% one-year arrhythmia-free survival [165] Furthermore, for persistent AF requiring substrate modification beyond PVI, such as posterior wall isolation, PFA achieved high rates of acute block and 12-month success without the attendant risks of esophageal or phrenic nerve injury characteristic of thermal approaches [175]. Collectively, these data from varied clinical scenarios position PFA as a versatile and effective therapy, demonstrating a consistent safety and efficacy profile across a wide spectrum of patient and substrate complexity.

#### Comprehensive safety profile and systematic management

The central tenet of PFA’s safety profile is its foundational biophysical property, myocardium-selective electroporation, which confers a paradigm shift in procedural safety by minimizing thermal collateral injury. However, this novel energy source also introduces unique potential risks that, alongside general procedural hazards, necessitate a systematic and proactive management framework (Table 2). Table 2 provides a detailed summary of PFA-specific and general procedure-related adverse events, along with their proposed mechanisms, typical incidence, and recommended prevention and management strategies.

**Table 2:** Safety monitoring and management of rare PFA complications

Complication	Incidence (%)^9^	Prevention/Monitoring	Acute Management
Esophageal injury	0.0	ICE guidance; low-energy test pulses; limit posterior wall applications; biphasic pulses; no esophageal temperature monitoring or other management strategies needed	PPI therapy; endoscopic surveillance; conservative management
Persistent Phrenic nerve palsy	0.0	Diaphragm EMG/fluoroscopy; avoid SVC/RSPV proximity; biphasic pulses	Cessation of pulses; high-flow O₂; steroids if needed
Hemolysis-related acute renal failure	0.03	IV hydration; consider limiting PFA applications for complex lesions; saline hydration	IV hydration; urine alkalinization; temporary hemodialysis if AKI
Coronary spasm	0.14	Low-energy test pulses; continuous ECG/BP monitoring; pre-administration of nitroglycerin considered	Intracoronary nitroglycerin; angiography if needed
Silent cerebral emboli	3–11.1	ACT > 300 s; DW-MRI 48–72 h; microbubble Doppler; filters/aspiration	Neurology consult; serial neurocognitive testing
Pericardial Tamponade	0.36	ICE for TSP and positioning; contact-force monitoring	Pericardiocentesis; surgical repair if severe
Vascular complications	0.30	Ultrasound-guided access; careful sheath handling	Manual compression; surgical repair if needed
Death	0.03	Careful patient selection; appropriate complication management	Resuscitation; specific management based on cause

PFA-specific adverse events, while rare, are mechanistically distinct from those of thermal ablation. These range from clinically significant hemolysis (< 1%), a direct consequence of erythrocyte membrane electroporation that is mitigated by meticulous catheter handling and optimized anticoagulation [152, 159]; to coronary artery spasm (rare), an electrical phenomenon that mandates procedural preparedness with readily available intracoronary vasodilators [176]. Skeletal muscle stimulation is not a complication but an anticipated physiological effect, the management of which through effective neuromuscular blockade and precise R-wave synchronization is integral to procedural stability and success [154, 155]. Finally, asymptomatic microembolic events detected on MRI occur at rates comparable to or lower than those of thermal modalities, a finding consistent with PFA’s non-thrombogenic mechanism [177].

General procedural risks, including tamponade, stroke, and vascular access complications, occur at rates consistent with or favorable to contemporary ablation benchmarks. A comprehensive safety paradigm is therefore multifaceted, integrating: (1) rigorous preprocedural planning with high-resolution imaging to anticipate anatomical challenges; (2) meticulous intraprocedural monitoring, using continuous ICE to verify catheter-tissue apposition and provide early detection of complications; (3) strict adherence to standardized protocols for anticoagulation, neuromuscular blockade, and R-wave synchronization [178–180]; (4) structured operator training and credentialing programs to navigate the technology’s learning curve; and (5) systematic post-procedural surveillance to monitor for delayed events.

Ultimately, the long-term stewardship of this technology depends on robust post-market surveillance. The establishment of centralized, multi-institutional adverse event registries is essential for promoting transparency, enabling sophisticated safety analyses, and establishing evolving benchmarks. Such a framework ensures that PFA’s favorable safety profile is not only preserved but is continuously monitored and optimized as clinical experience expands [181, 182].

### Other emerging device and pharmacologic strategies

Beyond PFA, the therapeutic armamentarium for AF is diversifying along two complementary axes: device- and procedure-based innovations that improve lesion delivery and substrate access, and pharmacologic/biologic strategies aimed at modifying the arrhythmogenic substrate or preventing disease progression. On the device side, advances are converging toward higher procedural precision and better substrate characterization. Robotic catheter-control and catheter-stabilization systems reduce operator-dependent variability and may improve reproducibility in complex left atrial anatomies. High-density electroanatomic mapping integrated with imaging and machine-learning algorithms enhance identification of non–pulmonary vein drivers and regions of fibrotic remodeling, permitting targeted, substrate-guided lesion sets rather than empiric extensive ablation [183, 184]. Alternative energy sources, focal laser, high-intensity focused ultrasound (HIFU), and chemical ablation of the vein of Marshall, offer distinct biophysical mechanisms that can be exploited for specific substrates or anatomic niches; epicardial and hybrid endo-epi approaches address deep or epicardial drivers that elude endocardial-only strategies [185–189]. LAA occlusion has matured as a device-based option for stroke prevention in patients unsuitable for long-term anticoagulation and increasingly features in integrated AF management pathways [190–192].

On the pharmacologic and biologic front, the field is shifting from symptomatic antiarrhythmic suppression to “upstream” and disease-modifying interventions. RAAS blockade [193–195], mineralocorticoid receptor antagonists, and agents targeting profibrotic signaling pathways have demonstrated variable efficacy in limiting atrial fibrosis and AF burden in mechanistic and selected clinical studies; anti-inflammatory therapies, like colchicine in perioperative contexts, and metabolic modulators, including emerging evidence for SGLT2 inhibitors, are being evaluated for AF prevention and post-ablation recurrence reduction [196–198]. Novel antiarrhythmic molecules with improved safety, as well as targeted molecular therapies, gene editing, RNA-based modulation, and cell therapy, represent longer-term prospects for substrate modification but remain largely preclinical or in early clinical evaluation. Importantly, the next step in innovation is rational combination: integrating substrate-modifying pharmacotherapies with precision ablation to improve lesion durability and reduce recurrence. Adoption of these technologies must be driven by mechanistic plausibility and comparative clinical evidence; pragmatic randomized trials, standardized outcome metrics, and health-economic analyses are required to define which patients benefit most and how innovations should be incorporated into guideline-based care.

## Advantages and limitations of major AF therapies

Within contemporary AF care frameworks that prioritize symptom relief, stroke prevention, and mitigation of disease progression, therapeutic selection is shaped by symptom burden, atrial remodeling, comorbidities, access, and patient preferences, and should be delivered through shared decision-making [22, 23]. In general, rate control alleviates palpitations and prevents tachycardia-induced cardiomyopathy but does not modify the arrhythmogenic substrate. AADs provide a non-invasive route to rhythm control, although long-term efficacy is modest and constrained by cumulative toxicities. Catheter ablation targets the triggers and sustaining substrate, most commonly via PVI, and improves arrhythmia freedom and quality of life in appropriate candidates, with energy-specific trade-offs in efficacy, efficiency, and safety among RF, CBA, and PFA [23, 84–89, 100–102]. Table 3 summarizes the comparative efficacy, advantages, principal limitations, typical candidates, and key safety considerations for the major therapeutic modalities for AF and can be used as a concise clinical reference.

**Table 3:** Comparative overview of major AF therapies: strengths, limitations, and use contexts

Modality	Efficacy	Key Advantages	Principal Limitations	Optimal Candidates	Key Safety Considerations
Rate Control	Symptom control; no rhythm restoration	• Widely accessible • Prevents tachycardia-mediated CM • β-blockers beneficial in CAD/HFrEF	• No sinus rhythm restoration • Limited impact on QoL • No effect on AF progression	• Older/frail patients • Minimal symptoms • High comorbidity burden • Unsuitable for rhythm control	• Bradycardia/hypotension • Negative inotropy • Digoxin toxicity
AADs	1-yr efficacy: 30–60% (varies by drug/substrate)	• Non-invasive • Restores/maintains SR • Amiodarone: broad substrate efficacy • Class Ic: safe in normal hearts	• Modest long-term efficacy • Proarrhythmia risk • Extracardiac toxicities • Multiple contraindications	•Paroxysmal AF: First-line • Bridge to ablation • Non-invasive preference • Temporary procedure contraindications	• QT prolongation • Organ toxicity • Drug–drug interactions • Regular monitoring required
RF Ablation	Paroxysmal: 70–80% at 1 yr Persistent: 50–60% at 1 yr (single procedure, off AADs)	• Versatile lesion creation • Addresses non-PV triggers • Substrate modification possible • Large evidence base • HPSD ↓ procedure time	• Thermal collateral injury risks • Lesion gaps → recurrence • Learning curve • Mixed benefit of extensive substrate ablation in persistent AF	• Paroxysmal/persistent AF with symptoms despite AADs • Complex anatomy • Non-PV triggers • Redo procedures	• Esophageal injury • Phrenic nerve palsy • Tamponade (0.8–1.3%) • PV stenosis
Cryoballoon Ablation	Paroxysmal: Non-inferior to RF (≈70–80% at 1 yr) Persistent: Similar to RF	• Single-shot PVI • Standardized workflow • Reproducible outcomes • Shorter learning curve • Procedural efficiency	• PVI-centric • Rigid catheter → access challenges • Efficacy in persistent AF requires adjunctive methods	• Paroxysmal AF • Centers prioritizing standardization/efficiency • Early operator experience	• Phrenic nerve palsy (2–6%) • Esophageal effects (transient) • PV stenosis • Vascular complications
PFA	Paroxysmal/persistent: Non-inferior to thermal (early data) Durability: maturing (12-mo excellent)	• Myocardium-selective • Extremely low esophageal/phrenic injury • Rapid procedures (30–60 min) • Promising in persistent AF • No general anesthesia needed	• Long-term durability (> 2–5 yrs) pending • Dosing not standardized across platforms • Access/cost variability • Rare hemolysis/coronary spasm	•Broad AF populations • High risk for thermal injury • Centers emphasizing safety/efficiency • Redo post-thermal failure	• Coronary spasm • Hemolysis • Standard anticoagulation • Catheter/guidewire handling

A rate-control strategy remains pragmatic for older patients with mild symptoms, high comorbidity burden, or those unsuited to invasive rhythm therapies. Beta-blockers, non-dihydropyridine calcium channel blockers, and digoxin can be tailored to activity levels and left ventricular function; beta-blockers are particularly advantageous in patients with coexisting coronary artery disease or HFrEF [22, 23, 79–81]. However, rate control does not restore sinus rhythm or reverse atrial remodeling. Some patients remain limited by exercise intolerance despite resting heart-rate targets, and there is no direct effect on slowing progression from paroxysmal to persistent AF [22, 23, 57]. In contrast, AADs are appropriate as a non-invasive first step for rhythm control in early paroxysmal AF, as a bridge to ablation, or when procedures are contraindicated. Flecainide and propafenone are suitable for patients without structural heart disease, while dofetilide and dronedarone can be considered under specific conditions, and amiodarone is effective across a broad range of substrates [82–85]. The principal constraints are a 1-year sinus rhythm maintenance rate typically around 30–60%, risks of proarrhythmia, and extracardiac toxicities like thyroid, pulmonary and hepatic with amiodarone. Dronedarone is contraindicated in symptomatic heart failure and permanent AF, and non-dihydropyridine calcium channel blockers are not appropriate for HFrEF [22, 23, 82–85].

Catheter ablation is associated with superior rhythm outcomes and quality-of-life improvements versus AADs in selected patients, with acceptable major complication rates when performed with evidence-based periprocedural anticoagulation and standardized workflows [24, 85]. RF ablation, including point-by-point and HPSD strategies, offers the greatest procedural versatility for complex anatomy, non-pulmonary vein triggers, substrate modification, and redo procedures; HPSD shortens procedure duration while maintaining efficacy [83–88, 99–101]. Limitations include risks of thermal collateral injury, a meaningful learning curve, and variable lesion durability influenced by gaps and tissue heterogeneity. In persistent AF, incremental benefit from extensive linear or substrate ablation beyond PVI has been inconsistent [28, 47, 51, 94–99]. CBA streamlines PVI with reproducible “single-shot” applications, a shorter learning curve, and non-inferiority to RF in paroxysmal AF, improving workflow efficiency and inter-center consistency. Its PVI-centric design, however, limits flexibility for non-PV triggers or complex substrates. The dominant complication profile includes phrenic nerve palsy and transient esophageal effects; in persistent AF, PVI-centric cryo outcomes are broadly similar to RF but generally require adjunctive techniques for non-PV targets [25, 28, 104, 109, 110].

PFA employs irreversible electroporation to achieve relatively myocardium-selective non-thermal ablation, demonstrating a favorable balance of speed, efficiency, and safety, especially a very low incidence of esophageal injury, phrenic nerve injury, and pulmonary vein stenosis, across registries and randomized trials. ADVENT showed non-inferior efficacy versus thermal energy with fewer major complications, and studies such as SPHERE Per-AF in persistent AF reported competitive outcomes alongside strong esophageal safety signals. Long-term durability data beyond 2–5 years are still maturing, inter-platform dosing and catheter designs are not yet fully standardized, and rare but distinctive safety signals require protocolized prevention and management strategies, adequate heparinization, catheter/guidewire handling, availability of nitrates [147, 162, 163, 165, 199]. Adoption is further influenced by local access and cost structures.

Therapy matching benefits from phenotype-based decision-making. Rate control is often preferred in older, frail patients with minimal symptoms and multiple comorbidities. For symptomatic paroxysmal AF in patients favoring early rhythm control, catheter ablation can be considered first-line, with energy selection tailored to operator expertise, safety priorities, and anatomical complexity: RF is well suited to individualized lesion sets and redo cases; CBA is optimal where standardized PVI and workflow reproducibility are priorities; and PFA is particularly attractive when minimizing thermal collateral injury is paramount or procedural efficiency is emphasized. In persistent AF, routine expansion of ablation beyond PVI does not uniformly improve outcomes; decisions should be anchored in the degree of remodeling, AF duration, heart failure status, and left atrial size, prioritizing high-quality PVI and evidence-based adjuncts. In patients with HFrEF, coupling rhythm control, especially ablation, with optimized heart failure therapy may yield greater functional and prognostic benefits. AADs retain an important role when invasive therapy is not feasible or desired, or as a bridge to ablation. For patients willing to pursue invasive therapy with the primary goals of symptom relief and quality-of-life improvement, ablation frequently provides more durable rhythm control.

Collectively, advances in evidence and technology are shifting AF management from one-size-fits-all approaches toward phenotype-guided, workflow-standardized, and outcome-verified strategies. Rate control ensures safety and comfort in selected populations; AADs offer a non-invasive option in early or transitional phases; and catheter ablation, leveraging complementary strengths of RF, cryo, and PFA, constitutes the central tool to achieve durable rhythm control and sustained quality-of-life gains. As longer-term PFA data, inter-platform dose standardization, and refined strategies for complex substrates emerge, energy selection will become increasingly individualized and evidence-driven.

## Conclusion

AF remains a prevalent and multifaceted clinical challenge that demands integrated strategies spanning mechanistic research, refined diagnostics, and tailored therapeutics. Stroke prevention and symptom-directed rate or rhythm control continue to be the cornerstones of management, while catheter ablation has reshaped rhythm control paradigms. Conventional thermal ablation has delivered meaningful clinical benefit but is constrained by incomplete lesion durability and collateral injury risk, motivating the development of novel, more selective technologies. PFA emerges as a promising nonthermal modality that offers myocardium selective lesioning and favorable procedural metrics, yet definitive conclusions regarding long-term durability, comparative effectiveness, optimal dosing, and standardized workflows await rigorous multicenter randomized trials and comprehensive registries. Beyond energy source innovation, progress in high-resolution imaging and electroanatomic mapping, the discovery and validation of biomarkers, and upstream pharmacologic approaches will be essential to refine substrate characterization and enable truly personalized interventions. Special patient groups including those with heart failure, valvular disease, advanced age, or multiple comorbidities require focused study to delineate who benefits most from invasive strategies. Priority research actions include large-scale randomized comparisons, systematic safety and durability surveillance, consensus on procedural standards and training, health economic analyses, and coordinated international registries to capture real-world outcomes. Through sustained multidisciplinary collaboration, iterative technology evaluation, and evidence-driven implementation, the field can advance toward more durable, safer, and individualized care for patients with AF.

## Data Availability

Data sharing is not applicable to this article as no new data were created or analyzed in this study. All information reviewed is available in the cited publications.
